# Matrix Mapping on Crossbar Memory Arrays with Resistive Interconnects and Its Use in In-Memory Compression of Biosignals

**DOI:** 10.3390/mi10050306

**Published:** 2019-05-07

**Authors:** Yoon Kyeung Lee, Jeong Woo Jeon, Eui-Sang Park, Chanyoung Yoo, Woohyun Kim, Manick Ha, Cheol Seong Hwang

**Affiliations:** Department of Materials Science and Engineering at Seoul National University, Seoul 08826, Korea; greense9@snu.ac.kr (Y.K.L.); wjd2153@snu.ac.kr (J.W.J.); euispark@snu.ac.kr (E.-S.P.); cyyoo0117@snu.ac.kr (C.Y.); kimkwh@snu.ac.kr (W.K.); manick.ha@snu.ac.kr (M.H.)

**Keywords:** resistive memory, crossbar, in-memory computing, analogue computing, matrix-vector multiplication, ECG

## Abstract

Recent advances in nanoscale resistive memory devices offer promising opportunities for in-memory computing with their capability of simultaneous information storage and processing. The relationship between current and memory conductance can be utilized to perform matrix-vector multiplication for data-intensive tasks, such as training and inference in machine learning and analysis of continuous data stream. This work implements a mapping algorithm of memory conductance for matrix-vector multiplication using a realistic crossbar model with finite cell-to-cell resistance. An iterative simulation calculates the matrix-specific local junction voltages at each crosspoint, and systematically compensates the voltage drop by multiplying the memory conductance with the ratio between the applied and real junction potential. The calibration factors depend both on the location of the crosspoints and the matrix structure. This modification enabled the compression of Electrocardiographic signals, which was not possible with uncalibrated conductance. The results suggest potential utilities of the calibration scheme in the processing of data generated from mobile sensing or communication devices that requires energy/areal efficiencies.

## 1. Introduction

Emerging classes of mobile electronic devices offer attractive capabilities for real-time analytics of the physical world through the connection to central computing systems. One of the critical challenges in this emerging Internet of Things (IoT) is the instantaneous extraction of relevant information from the abundant data with the limited power and communication bandwidth for data transmission. This challenge demands smart components on the edge of the mobile devices that can filter, compress, or classify the data outputs onsite [1,2,3,4]. This pre-processing needs to be extremely power efficient and quick to handle the large volume of data continuously generated from the surrounding world.

A subset of the processing operations can be categorized as a linear transformation which can be expressed as a matrix-vector multiplication (MVM). The MVM can be performed in an analogue domain using a resistive memory crossbar array by storing the matrix values as the conductance of the memory cell. The operation can take a constant time complexity (O(1)), and be energy efficient owing to the functional integration of the processing and memory units [5,6,7]. The scalability of the crossbar structure down to $4F^{2}$ (F: feature size of a technology node) is also beneficial for the device miniaturization. Envisioned applications include linear equation solver and training of or inference on neural networks as demonstrated recently [1,7,8,9,10,11]. 

Prior studies have shown that the throughputs per area and the energy efficiency can exceed today’s von Neumann computing scheme, but computational accuracy remained as a non-trivial challenge for high-precision analogue-based MVM. In device levels, output errors can be originated from the variations of the electrical characteristics between the cells, non-linear current-voltage relationship, and stochasticity in resistance switching process. Separate from the efforts in development of the reliable devices, it is also important to optimize the conductance mapping scheme using realistic crossbar arrays. Finite conductivity of interconnecting wire has been suggested as one of the important factors causing errors in the crossbar-based MVM [9,12]. Empirical calibration methods that are based on the comparison between the desired output and real measurements have shown to improve the accuracy level although the origin of the discrepancy of the measurement values was not clearly identified [1]. To overcome the limitation of such hardware-based methods, model-based theoretical analysis attempted more systematic approach to understand the computational error [9,12]. Hu et al. first introduced a comprehensive crossbar array model for MVM, and applied it to the training of neural network for pattern recognition [9]. This simulation-based optimization of the conductance minimizes the time and power consumption to post-process the outputs and provides explanation for the computational outputs with given circuits.

This work implemented a mapping algorithm of memory conductance for MVM using a crossbar model with finite wire resistance, and analyzed the calibration performance for the compression of electrocardiographic (ECG) signals. An iterative software simulation calculates the matrix-specific local junction voltages at each cross-point, and calculate the ratio between the junction voltages and input voltage applied from the source. The ratio becomes a calibration factor to update the memory conductance to systematically compensates the voltage drop. The results indicate that the calibration factors both depend on the location of the junctions and matrix structure. This correction enabled the in-memory compression of ECG signals whose reconstruction error is comparable to the double precision calculation. The findings suggest a possible route to overcome difficulties in analogue computing in realizing diverse edge computing devices for onsite data processing.

## 2. Methods 

### 2.1. Calibration Factor for Matrix Mapping on Proposed Crossbar Model

**Figure 1:**
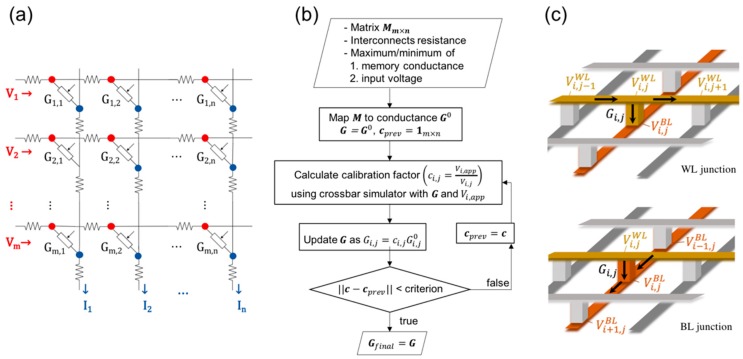
(**a**) Simulation model for resistive memory crossbar array with finite conductance of interconnects. (**b**) Conductance calibration algorithm for mapping of an m×n matrix using a crossbar simulator. (**c**) Local currents at word lines (WL) and bit line (BL) junctions in accordance with Kirchhoff’s law.

Figure 1a shows a schematic representation of the crossbar model that includes interconnection line resistance to calculate the local potential at each cross-point. The model incorporates both the cell-to-cell resistance and the access resistance from a voltage source to the first column/row metal lines. The analogue-based MVM using a crossbar array assuming an ideal behavior has the current output from the column (or bit) line (BL) as follows.

(1) $$I_{j}^{ideal}=G_{1,j}V_{1,app}+\cdots +G_{m,j}V_{m,app}$$

Here, $I_{j}^{ideal}$ is the current output from j^th^ BL. $G_{i,j}$ is the conductance of memory cell located at a crosspoint of the i^th^ word and the j^th^ bit lines. The conductance ($G_{i,j}$) represents a linear-transformed matrix element to map the matrix values within the range of the achievable conductance of the device. $V_{i,app}$ is input voltage to the i^th^ word lines (WL). (BLs are assumed to be grounded.) Equation (1) holds true only if the series resistance of the interconnection wires is negligible. Considering the resistivity of conventional metal wires (ρ = 10^−^^8^ to 10^−7^ Ω·m), the resistance between the nearest cells (R=ρ·F/(F·d), F: feature size, d: metal thickness) ranges from 10^0^ to 10^1^ Ω when d is assumed ~10 nm. The wire resistance may further increase due to lower density caused by vapor deposition. For a 4F^2^ crossbar structure, the interconnect resistance between two adjacent cells can be estimated to be ~4.53, 2.97, and 1.55 Ω under 16 nm, 22 nm, and 32 nm technology node, respectively, according to the International Technology Roadmap for Semiconductors 2013 [12]. Simple calculation estimates the voltage drop can be a significant source of errors considering the realistic conductivity of the resistive memories. For example, if we assume ~100 by 100 bits of crossbar arrays and 0.1 to 1 mA total current along the word line, iR drop at the end of the word line can be 0.01 to 0.1V. (e.g., 0.1–1 mA × R(cell-cell) × 100 → 0.01–0.1 V). In this realistic case, the current output needs to be modified as

(2) $$I_{j}^{real}=G_{1,j}V_{1,j}+G_{2,j}V_{2,j}+\cdots +G_{m,j}V_{m,j}$$

instead of Equation (1) with $V_{i,app}$ terms to conform with the Ohm’s law. Here, $V_{i,j}$ is the local junction potentials across the memory cell at (i,j) crosspoint. Since $V_{i,j}$ is not guaranteed to be equal to the applied voltage to the i^th^ WL due to voltage drop, $I_{j}$ becomes small compared to the ideal case as observed in previous studies [1,9]. 

One way to compensate the smaller current output can be the increase of the conductance level of the memory according to the local voltage drop. If the voltage drop for arbitrary WL and BL input voltages can be estimated, the conductance of the memory can be set as

(3) $$G_{i,j}^{\prime }=G_{i,j}\frac{V_{i,app}}{V_{i,j}}$$

instead of $G_{i,j}$. With the calibrated conductance ($G_{i,j}^{\prime }$), the current outputs become the ideal current as follows.

(4) $$I_{j}^{real}=G_{1,j}\frac{V_{1,app}}{V_{1,j}}\cdot V_{1,j}+\cdots +G_{m,j}\frac{V_{m,app}}{V_{m,j}}\cdot V_{m,j}=I_{j}^{ideal}$$

Thus, the ratio ($V_{i,app}/$$V_{i,j}$) can be considered as a calibration factor for the memory conductance for in-memory MVM when the junction potential deviates from the applied voltage. There can be other approaches that use equilvalent conductance terms multiplied by the applied voltage to describe the measured current. This approach may be useful if measurement data are available and the calibration algorithm to drive the real current to the ideal one is developed. Yet, the current work is more focused on the calibration based only on theoretical model circuits without requirement for any real measurements.

### 2.2. Iterative Calibration Based on Crossbar Simulation

An iterative algorithm was developed to progressively increase conductance values based on the simulated $V_{i,j}$ at individual junctions. Figure 1b summarizes the procedure of the calibration process. Through the iterations, ${V_{i,j}}^{\prime }s$ are updated by solving the 2mn Kirchhoff’s relations (mn WL junctions + mn BL junctions) that need to be simultaneously satisfied with given memory conductance and the voltage inputs [13]. Figure 1c, for example, illustrates the local currents on the WL junction that follow the equation below.

(5) $$G_{w}\left(V_{i,j}^{WL}-V_{i,j-1}^{WL}\right)=G_{i,j}\left(V_{i,j}^{BL}-V_{i,j}^{WL}\right)+G_{w}\left(V_{i,j+1}^{WL}-V_{i,j}^{WL}\right)$$

Here, $G_{w}$ is a cell-to-cell conductance, and $V_{i,j}^{WL}$ and $V_{i,j}^{BL}$ are voltages at (i,j) crosspoint on WL and BL, respectively. 2mn Kirchhoff’s equations can be arranged in a simple matrix form whose details are described in the Appendix A. Since the calibrated conductance ($G_{i,j}^{\prime }$) is higher than the previous conductance ($G_{i,j})$, the overall current increases, and the voltage drops need to be recalculated with this new $G_{i,j}^{\prime }$ by the next iteration of the simulation. The iteration is repeated until the conductance (or $V_{i,app}/V_{i,j}$ ratios) converge, and the final ratios determine the conductance level of the memory to represent the arithmetic matrix elements. The simulation code is implemented in MATLAB and each iteration takes ~1 sec with single 3.5 GHz Intel Core i7 for 64 × 64 crossbar arrays. The calibration factors were converged after 10 to 20 iterations depending on the cell-to-cell resistance and termination criteria. The runtime and error depend on the termination criteria, and assumed to be a similar level to the previous report [9].

## 3. Results and Discussion

The in-memory MVM can be used for low-power data processing, such as compression or high- or low-pass filtering. Here, as an example, the discrete wavelet transform (DWT) matrix is mapped to the final memory conductance ranging from 0.01 to 70 μS [14,15]. The cell-to-cell resistance (R) and the access resistance from the voltage source to the crossbar are assumed to be 1 Ω and 100 Ω, respectively. Larger R (10 Ω) is also studied for comparison. Voltages are supplied from the left for WLs and the bottom for BLs. For the calculation of the voltage drops at each junction, the supply voltage of 0.1 V was assumed for all WLs. (The calibration factors were insensitive to the voltage (0.1 to 0.5 V) since $V_{i,j}$ ~ $V_{i,app}-iR$ where iR varies approximately with the same factor as $V_{i,app}$). The operation parameters were set to be consistent with the practical values reported in the previous PRAM-based studies [7].

**Figure 2:**
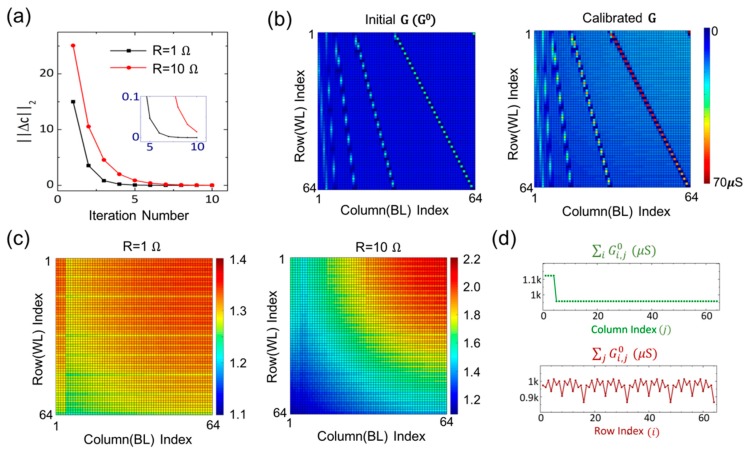
Conductance mapping of 64 × 64 matrix for discrete wavelet transform (DWT). (**a**) Convergence of calibration factors though the iterations for 1 Ω and 10 Ω cell-cell resistance. (**b**) Colored map of cell conductance of a crossbar before/after calibration. (R = 10 Ω). (**c**) Matrix-specific calibration factors at individual cross-points for R = 1 Ω (left) and R = 10 Ω (right). (**d**) Conductance sum of each column (top) or row (bottom) of the initial conductance.

Figure 2 presents the simulation results of the conductance mapping of 64 × 64 DWT matrix using biorthogonal filters with 4-level of decomposition. Figure 2a describes the change in the calibration factors through the iteration represented by the 2-norm of the difference matrix. The conductance is quickly converged, and the norm values less than $10^{-4}$ were achieved after 10 cycles (R = 1 Ω) and 16 cycles (10 Ω). Figure 2b compares the initial conductance ($G_{i,j}^{0}$) and final conductance for R = 10 Ω case. Figure 2c plots the final calibration factors to visualize the voltage drop across the crossbar. (R = 1 Ω (left), 10 Ω (right)) Calibration factors range from 1.1 to 1.4 for 1 Ω case, and 1.1 to 2.2 for 10 Ω case. 10 Ω resistance shows larger dependency of the calibration factor on the distance from the voltage source. The location dependency of the calibration factors implies that the effect of possible fluctuation in the resistance of nanoscale wires can be averaged over the long distance from the voltage source for the junctions with large calibration factors. The colormaps also reveal the large values for the first four columns and small values for every four rows. As depicted in Figure 2d, the calibration factors reflect the matrix structure. The conductance sum ($\sum_{i}G_{i,j}^{0}$) is large for the first four columns, which results in a large current gathered along the four BLs. For the same reason, the small conductance sum ($\sum_{j}G_{i,j}^{0}$) for every four rows result in small overall current along the WLs: thus, smaller calibration factors. This variation in the overall current along the metal line causes different level of iR drop, resulting in matrix-dependent calibration factors.

**Figure 3:**
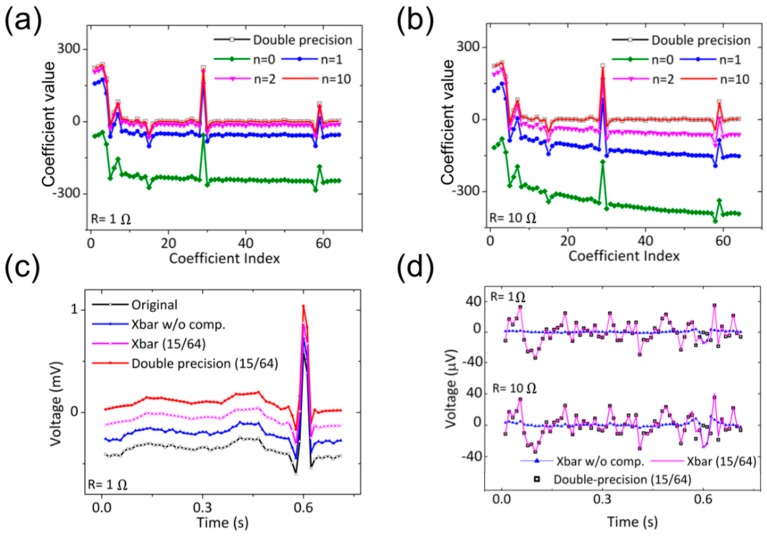
Electrocardiographic (ECG) signal compression using in-memory computing. (**a**,**b**) Coefficients of ECG signal after DWT using crossbar (Xbar) conductance determined by simulation. n: iteration number of simulation for conductance calibration. (**a**) R = 1 Ω. (**b**) 10 Ω. (**c**) Reconstruction of ECG from the coefficients. Compression ratio = 15/64. (**d**) Reconstruction error.

Figure 3 summarizes the effect of the conductance calibration on the data compression and reconstruction performance. Rescaled ECG signals from the MIT-BIH database were applied as the input voltage (0–0.3 V) for DWT [16]. Figure 3a,b show the coefficients of the DWT converted from the simulated currents from the BLs for R = 1 Ω and 10 Ω, respectively. The black squares present the exact coefficients calculated in double-precision (64 bits), and the green diamond lines present the simulated coefficients with the initial memory conductance before calibration. The negatively shifted values of the simulated coefficients result from the small currents due to the voltage drop along the resistive metal interconnects. This shift fails the threshold-based compression of data where the small coefficients are cut off based on their absolute quantity (distance from zero). The larger negative slope in Figure 3b compared to Figure 3a reflects a severe reduction in current outputs for the columns located far from the voltage source due to the larger R (10 Ω). The other lines in the figures show the coefficients calculated with the calibrated memory conductance at different stages of iteration. The red lines in Figure 3a,b show that the fully calibrated coefficients well match to the exact values for both R values. The 2-norms of the difference between the exact and the experimental coefficient vectors were 4.2 (1 Ω) and 8.6 (10 Ω), and the maximum difference were 3.5 (1Ω) and 7.2 (10 Ω) at the peak of the coefficient (exact coefficient value: 224.8, index: 29). Figure 3c shows the reconstructed ECG signals using the calibrated coefficients. (ECG signals were vertically shifted for visibility of individual lines.) The magenta line shows the reconstructed signals from the 15 largest exact coefficients out of 64. By filtering of the small coefficients, the noise in the original signal was removed as the case with exact coefficients. Figure 3d plots the error of the reconstructed signal. The reconstructed signal-to-noise ratios, defined as $20{log}_{10}({\left|\left|x\right|\right|}_{2}/{\left|\left|x-\hat{x}\right|\right|}_{2})\;$(x: original ECG, $\hat{x}$: reconstructed ECG), were 28.2/43.4 (1 Ω) and 27.8/37.1 (10 Ω) with/without cut-off, respectively, compared to 28.3 for the reconstruction using 15 largest exact coefficients. 

## 4. Conclusions

A conversion algorithm of a matrix to conductance was proposed in a crossbar memory array when the metal interconnects have finite conductance. The iterative simulation systematically compensates for the voltage drop along the interconnects by increasing the memory conductance. The calibration enables in-memory data compression. Considering the power limit in healthcare-related mobile devices, the proposed real-time compression using a memory crossbar can have potential as pre-processing units in such devices for diagnosis/therapeutic purposes.

## Figures and Tables

**Figure A1:**
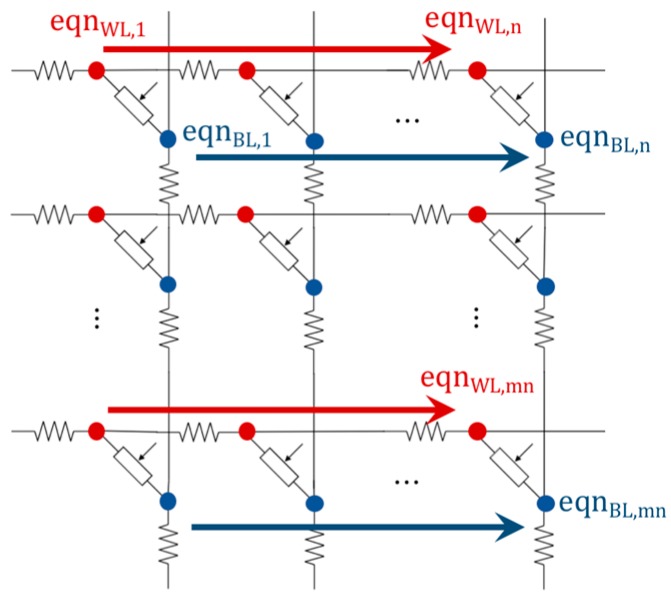
Kirchhoff’s law produces 2*mn* equations.

## Appendix A

The crossbar model aims to calculate junction potentials at each cross-point. Since we can build one Kirchhoff’s equation for each junction, 2mn relations (Figure A1, mn junctions on WL+ mn junctions on BL) need to be simultaneously satisfied with given memory resistances and the applied WL and BL applied potentials. Here, $G_{w}$ and $G_{i,j}$ are the wire and memory conductance, and $V_{i,j}^{WL}$ and $V_{i,j}^{BL}$ are the local voltages at the junctions in a real system with finite conductance of the interconnects.

(A1) $$\left(WL,\;(i,j\right))\;\;\;\;\;\;\;\;\;G_{w}\left(V_{i,j}^{WL}-V_{i,j-1}^{WL}\right)-G_{i,j}\left(V_{i,j}^{BL}-V_{i,j}^{WL}\right)-G_{w}\left(V_{i,j+1}^{WL}-V_{i,j}^{WL}\right)=0$$

(A2) $$\left(WL,\;j=1\right)\;\;\;\;G_{i,access}^{WL}\left(V_{i,1}^{WL}-V_{i,applied}^{WL}\right)-G_{i,1}\left(V_{i,1}^{BL}-V_{i,1}^{WL}\right)-G_{w}\left(V_{i,2}^{WL}-V_{i,1}^{WL}\right)=0$$

(A3) $$\left(WL,\;j=n\right)\;\;\;\;\;\;\;G_{w}\left(V_{i,n}^{WL}-V_{i,n-1}^{WL}\right)-G_{i,n}\left(V_{i,n}^{BL}-V_{i,n}^{WL}\right)=0$$

(A4) $$\left(BL,\;\left(i,j\right)\right)\;\;\;\;\;\;\;\;\;G_{w}\left(V_{i+1,j}^{BL}-V_{i,j}^{BL}\right)-G_{i,j}\left(V_{i,j}^{BL}-V_{i,j}^{WL}\right)-G_{w}\left(V_{i,j}^{BL}-V_{i-1,j}^{BL}\right)=0$$

(A5) $$\left(BL,\;i=m\right)\;\;G_{j,access}^{BL}\;\left(V_{j,applied}^{BL}-V_{m,j}^{BL}\right)-G_{m,j}\left(V_{m,j}^{BL}-V_{m,j}^{WL}\right)-G_{w}\left(V_{m,j}^{BL}-V_{m-1,j}^{BL}\right)=0$$

(A6) $$\left(BL,\;i=1\right)\;\;\;\;\;\;\;G_{w}\left(V_{2,j}^{BL}-V_{1,j}^{BL}\right)-G_{i,j}\left(V_{1,j}^{BL}-V_{1,j}^{WL}\right)=0$$

When the equations are arranged in the order as described in Figure A1, the equations can be simplified as the following matrix formulation:

(A7) $$A_{mn\times mn}v_{WL}+B_{mn\times mn}v_{BL}=E_{WL}\;(\mathrm{for}\;\mathrm{WL}\;\mathrm{junctions})$$

(A8) $$C_{mn\times mn}v_{WL}+D_{mn\times mn}v_{BL}=E_{BL}\;(\mathrm{for}\;\mathrm{BL}\;\mathrm{junctions})$$

(A9) $$\left[\begin{array}{cc} A & B\\ C & D \end{array}\right]\left[\begin{array}{c} v_{WL}\\ v_{BL} \end{array}\right]=\left[\begin{array}{c} E_{WL}\\ E_{BL} \end{array}\right]$$

where

(A10) $$v_{WL,mn\times 1}={\left[V_{1,1}^{WL},V_{1,2}^{WL},\cdots ,V_{1,n}^{WL},V_{2,1}^{WL},\cdots ,V_{m,n}^{WL}\right]}^{T}={\left[v_{WL,i=1},v_{WL,i=2},\cdots ,v_{WL,i=m}\right]}^{T}$$

(A11) $$v_{BL,mn\times 1}={\left[V_{1,1}^{BL},V_{1,2}^{BL},\cdots ,V_{1,n}^{BL},V_{2,1}^{BL},\cdots ,V_{m,n}^{BL}\right]}^{T}={\left[v_{BL,i=1},v_{BL,i=2},\cdots ,v_{BL,i=m}\right]}^{T}.$$

(A12) $$E_{WL,mn\times 1}={\left[G_{1,access}^{WL}V_{1,app}^{WL},0,\cdots ,G_{2,access}^{WL}V_{2,app}^{WL},0,\cdots ,G_{m,access}^{WL}V_{m,app}^{WL},0,\cdots \right]}^{T}$$

(A13) $$E_{BL,mn\times 1}=-{\left[G_{1,access}^{BL}V_{1,app}^{BL},G_{2,access}^{BL}V_{2,app}^{BL},\cdots ,G_{n,access}^{BL}V_{n,app}^{BL}\;0,\cdots \right]}^{T}$$

Here, A and D are sparse matrices whose nonzero elements are the ones that are multiplied by the local potentials adjacent to the junction under consideration along the WL (for A) or BL (for D). For example, the Kirchhoff’s law on the (i,j) WL junction is described by

(A14) $$A_{\left(i-1\right)\times j+j^{th}\;row\;\;}v_{WL}+B_{\left(i-1\right)\times j+j^{th}\;row\;}v_{BL}=E_{WL,\left(i-1\right)\times j+j^{th}\;row}$$

The only nonzero elements of (i−1)×j+j^th^ row of A are j−1, j, j+1^th^ elements of the row. B and C are mn×mn diagonal matrices related to the conductance of the resistive memory to describe the currents flow through the memory layer. More details are available in [13] although the structure of the matrices A, B, C, D, $E_{WL}$ and $E_{BL}$ depends on the order of the Kirchhoff’s equations that correspond to the individual junctions.

For the simulation where all the applied potentials to the WL and BL are set, local potentials at the crossbar junctions can be obtained in two steps by solving the following two equations:

(A15) $$\left(B-AC^{-1}D\right)v_{BL}=E_{WL}-AC^{-1}E_{BL}$$

(A16) $$v_{WL}=C^{-1}\left(E_{BL}-Dv_{BL}\right)$$
